# Anti-tumor effects of Abnormal Savda Munziq on the transplanted cervical cancer (U27) mouse model

**DOI:** 10.1186/s12906-016-1458-5

**Published:** 2016-11-24

**Authors:** Zuhragul Omarniyaz, Yang Yu, Tao Yang, Lianlian Shan, Weiwei Miao, Renaguli Reyimu, Halmurat Upur, Ainiwaer Aikemu

**Affiliations:** 1 Department of pharmaceutical analysis, Xinjiang Medical University, Urumqi, 830011 China; 2 Uyghur Medical College, Xinjiang Medical University, Urumqi, 830011 China; 3 Central Laboratory of Xinjiang Medical University, Urumqi, 830011 China

## Abstract

**Background:**

Abnormal Savda Munziq (ASMq), a traditional uyghur medicine, has shown anti-tumour properties in vitro. it was showed that total flavonoids of ASMq could inhibit the proliferation and enhance the antioxidant ability of human cervix cancer HeLa cell. This study attempts to confirm these effects on the transplanted cervical cancer (U27) mouse model in vivo.

**Methods:**

Forty eight Kunming mice were randomly divided in to six groups: normal control group (Control group), U27 tumor model group (Model group), cyclophosphamide administration group (CTX group),low-dose ASMq group (ASMq.L group), medium-dose ASMq group (ASMq.M group), and high-dose ASMq group (ASMq.H group). The five groups except normal control group transplanted with cervical cancer (U27) cells. We observed mice tumor inhibition rate and conducted the histopathological analysisUsing the western blot assay, the expression of TGF-β1 and TNF-α protein in transplanted cervical cancer U27 tumor tissue were detected.

**Results:**

The tumor inhibition rates of CTX group, ASMq.L group, ASMq.M group, and ASMq.H group were 72.21, 31.27, 60.53 and 51.94% respectively, has obvious antitumor effect. ASMq significantly promote the spleen tlymphocyte proliferation of transplanted cervical cancer U27 mice. Invasive growth and diffusion rate in tumor tissue were accelerate in the transplanted cervical cancer U27 model group. Tumor tissue necrosis of tumor cells are smaller in the medium, high dosage group. Compared with the U27 model group, the expression levels of TGF-β1 protein and TNF-α protein expression exhibited statistically significant decreased in the mice tumor tissues in the CTX administration group and the ASMq administration group.

**Conclusions:**

ASMq has some antitumor effects on U27 model mice in vivo, The effects are achieved not only by improving the immune function of U27 model mice, but also by inhibiting the expression levels of TGF-β1 protein while promoting the expression levels of TNF-α protein.

## Background

Cervical cancer is a high-incidence malignancy in women, it has been severely impacts the lives of women worldwide [1, 2]. According to literature, over 500,000 new cases occur globally every year, approximately half of them are fatal [3]. The morbidity and mortality rates of Xinjiang Uigur women are higher than those of other ethnic women in Xinjiang. In fact, the mortality rate ranks number 1 in Chinese minorities.

In the earliest Greco-Arab medical texts, *Hippocratic Corpus*, Hippocrates proposed a series of theorems based on liquids and their volumes. Hippocrates suggested that the human body is primarily comprised of four Hilits, or the “body fluids”, which includes blood (Kan), black bile (Savda), yellow bile (Safra), and phlegm. Each of these body fluids has its own unique color (red, black, yellow, and white), viscosity, and basic qualities. After the onset of disease, a person exhibits an excess of body fluids that disrupts the normal balance [4]. Uigur medicine, one of the three traditional types of Chinese medicine, originated from Greco-Arab medicine [5]. Uigur medicine also holds that the human body is comprised of four kinds of body fluids. Abnormal savda is considered the final product of all of body fluids in the presence of abnormal changes. In addition, in Uigur medicine, tumor development is related to the formation of abnormal savda. Since abnormal savda is thick in texture, it can easily result in biliary stasis if deposited in vessel walls, leading to cancer and other intractable diseases. Abnormal Savda Munziq (ASMq) is a natural compound prepared using ten types of herbs, including *Glycyrrhiza uralensis Fisch., Adiantum capillus-veneris L., Euphorbia humifusa Willd., Alhagi pseudalhagi Desv.* (as show in Table 1) [6]. In the previous studies, it was showed that total flavonoids of ASMq can inhibit the proliferation and enhance the antioxidant ability of human cervix cancer HeLa cell [7]. In addition, the Abnormal Savda Munziq had obvious inhibition effect on liver cancer and breast cancer, and the effect of Abnormal Savda Munziq was strong scavenging hydroxyl free radical, protecting DNA oxidative damage and inducing apoptosis [8–14].

**Table 1:** Plants used in Uyghur herbal preparation Abnormal Savda Munziq (ASMq)

Uyghur Name	Latin name	Family	Part used
Pirsiyavxan	*Adiantum capillus-veneris L.*	Adiantaceae	Whole plant
Kök tantak	*Alhagi pseudalhagi (Bieb.) Desv.*	Fabaceae	Branch secretion
Gavziban	*Anchusa italica Retz.*	Boraginaceae	Whole plant
Serbistan	*Cordia dichotoma G.Forst.*	Boraginaceae	Fruit
Yalmankülak	*Euphorbia humifusa Willd. Euphorbia maculata L.*	Euphorbiaceae	Whole plant
Arpabidiyan	*Foeniculum vulgare Mill.*	Apiaceae	Fruit
Qüqük buya	*Glycyrrhiza uralensis Fisch. ex DC.*	Fabaceae	Radix or rhizoma
Üstihuddus	*Lavandula angustifolia Mill.*	Lamiaceae	Aerial parts
Badrenjiboye hindi	*Melissa officinalis L.*	Lamiaceae	Whole plant
Qilan	*Ziziphus jujuba Mill.*	Rhamnaceae	Fruit
	*Glycyrrhiza inflata Batalin*	Fabaceae	Radix or rhizoma
	*Glycyrrhiza glabra L.*	Fabaceae	Radix or rhizoma

In this study, the anti-tumor effects of ASMq on U27 tumor model mice were investigated, providing an experimental basis for the clinical treatment of cervical cancer with the Uigur drug ASMq and contributing to the recognition of its anti-tumor effects on U27 tumors.

## Methods

### Animals and cell lines

A total of 48 healthy Kunming mice (24 male, 24 female) weighing 20 ± 2 g, were provided by the Xinjiang Medical University Experimental Animal Center (license number: SCXK (Xin) 2011-0004). The U27 cervical cancer cell lines were purchased from Wuhan University.

### Laboratory instruments and reagents

The analytical balance (Model: AL204) and electronic balance (Model: PL602S) were purchased from Shanghai Mettler-Toledo Instruments Co., Ltd. The -80 °C ultra-low temperature freezer was purchased from Haier, China. The low temperature refrigerated centrifuge (Model: FRESCO 17) and microplate reader (Model: MULTISKAN MK3) were purchased from Thermo. The shaker (Model: TS-8), vortex (Model: QL-861), thermostat (Model: GL-150B), and magnetic stirrer (Model: GL-3250C) were purchased from Kylin-Bell Lab Instruments Co., Ltd. The electrophoresis, membrane transfer device Tanon (Model: VE-180), and scanner (Model: MFC7450) were purchased from Brother. The Hypersensitivity ECL Chemiluminescence Kit (Cat. No.: B0018) and BCA Protein Assay Kit (Cat. No.: P0012) were purchased from Beyotime. β-Actin (Size: 0.2 ml) was purchased from Boster. The medical X-ray films (Specification: Super RX) were purchased from Fuji. TGF-β1 (Size: 100 μg, Item No: A1-29020) and TNF-α antibodies (Size: 100 μg, Item No: PA5-19810) were purchased from Thermo Scientific. ASMq was purchased from Xinjiang Cicon Habo Uighur Medicine Ltd. Corporation. cyclophosphamide (Batch number: 13122625) was purchased from Jiangsu Hengrui Medicine Co., Ltd. Saline (Batch number: 1311161) was purchased from Sinopharm Xinjiang Pharmaceutical Co., Ltd. Hematoxylin and Eosin Staining Kit, Matrigel (BD Biosciences, USA).

### Animal grouping

The 48 healthy Kunming mice were randomly divided into six groups of eight mice (four male, four female), including the normal control group, the U27 tumor model group, the CTX administration group, and the low-, medium-, and high-dose ASMq groups.

### Construction of the U27 tumor mouse model

The 40 mice in the groups other than the normal control group were used construct the transplanted cervical carcinoma (U27) tumor model. The following experimental method was implemented [15]. Ascitic fluid (milk white color) was drawn from the U27 Kunming mice 7 days after inoculation (intraperitoneal injection of 0.2 mL 1.0 × 10^7^ cfu/mL cells) and diluted to a concentration of 1.0 × 10^7^ cfu/mL with saline sterilized at high pressure. 0.2 mL of ascites suspension was injected into the left forelimb axilla of each mouse. The entire process was conducted under sterile conditions within 30 min. After 24 h, the drugs were administered according to body weight. 0.2 mL/10 g (equal amounts of) saline were administered to the control group and U27 tumor model group via gavage every day. In addition, the mice in the control group and U27 tumor were administered intraperitoneal injections of 0.2 mL/10 g saline every other day. The mice in the CTX administration group were administered 0.2 mL/10 g saline via gavage every day and an intraperitoneal injection of 30 mg/kg CTX every other day. The mice in the low-, medium-, and high-dose ASMq groups were administered 2, 4, and 8 g/kg ASMq, respectively, via gavage every day and 0.2 mL/10 g intraperitoneal injections of saline every other day. All of the mice were provided with food and water, weighed every day, and administered their respective medications via gavage for 10 continuous days.

### Sample preparation

The Kunming mice were euthanized after continuous gavage for 10 days. Then the tumor tissues of U27 and the thymus, spleen, liver, kidneys each mouse were extracted. The tumor tissues were dissected into two portions quickly, one was transferred into a frozen tube, and the other portion was fixed in 10% formalin and dehydrated for hematoxylin and eosin (HE) staining.

### Determination of the tumor inhibition rate and the spleen, thymus, liver, and kidney indexes

Tumor inhibition rate = [(the average tumor weight of the tumor model group - the average tumor weight of the treated group)/the average tumor weight of the tumor model group] × 100%. Thymus index = thymus weight (mg)/body weight (g). The spleen, liver, and kidney indexes were calculated similarly [16].

### Preparation and HE staining of the U27 tumor tissue histological sections

The 10% formalin-fixed, dehydrated U27 tumor tissues were embedded in paraffin and processed into 4 μm paraffin sections. After HE staining, the sections were cleared with xylene and mounted in neutral gum [17]. The cell morphology of U27 tumor tissue sample was carefully observed under an optical microscope.

### TGF-β1 and TNF-α protein expression in the U27 tumor tissues

A small amount of each tumor tissue sample was weighed into a homogenizer and combined with RIPA lysis buffer. After homogenization, the tissue samples were placed on ice in order to ensure thorough lysis. Then, each sample was transferred into a centrifuge tube and centrifuged at 4 °C pre-cold centrifuge at 12,000 r/min for 5 min. The supernatant was collected for protein concentration determination via the BCA method. First, 4 mL 10% resolving gel was prepared, and 4% stacking gel was quickly poured into the gel sandwich. The samples were gently loaded into the gel wells. The electrophoresis apparatus was connected to the power supply and run under constant voltage. First, the apparatus was set at 80 V, or approximately 8 V/cm, until each sample passed through the interface between the stacking gel and resolving gel. Then, the apparatus was set to 120 V until the dye reached an appropriate location. A PVDF membrane was soaked in 100% methanol for 2–3 min, then rinsed with water and Western transfer buffer twice for 2 min. The membrane was kept in the transfer buffer. Six layers of filter paper the same size as the gel were cut and soaked in the transfer buffer. The protein was transferred at 4 °C for 1 h. In addition, the membrane was soaked in the blocking buffer at room temperature and shaken slowly for 1 h. Then, the membrane was added to the primary antibody and diluted in the blocking buffer at 4 °C overnight. After being washed three times with the TBST buffer, the secondary antibody was added and allowed to react for 2 h. Then, the membrane was washed with the TBST buffer again in order to remove the free secondary antibody. Finally, the membrane was exposed in the dark. The average values were calculated after repeating this process three times.

### Data processing and analysis

All of the data was tested for normality and homogeneity of variance before further analysis. Statistic analysis was performed with SPSS17.0 One-way analysis of variance ANOVA and chi-square tests where experimental data was indicated as mean ± SD. The mean difference is considered significant at the *p*-value < 0.05 level.

## Results

### Effects of ASMq on the thymic, splenic, hepatic, and renal weights of the U27 tumor mice

Compared to the normal control group, the thymic, splenic, and renal weights of the U27 tumor model mice are all lower (*p* < 0.05); the thymic, splenic, hepatic, and renal weights of the CTX group are all decreased (*p* < 0.05); the splenic, hepatic, and renal weights of the low-dose ASMq group are all dropped (*p* < 0.05). In contrast, the splenic and hepatic weights of the medium-dose ASMq group are increased (*p* < 0.05), while the renal weight is decreased (*p* < 0.05). Furthermore, the thymic and hepatic weights of the high-dose ASMq group are both elevated (*p* < 0.05). Compared to the U27 tumor model group, the weights of all of the major organs in the CTX group are decreased (*p* < 0.05), while the thymic, splenic, and hepatic weights of the ASMq group are increased (*p* < 0.05). Compared to the CTX group, the weights of all of the major organs in the ASMq group are elevated (*p* < 0.05), as shown in Table 2.

**Table 2:** ASMq’s effect on the important organ weight of in each group mice (*n* = 8, $\overline{x}\pm s$)

Group	Dosage/(g/kg)	Thymus weight	Spleen weight	Liver weight	Kidney weight
Normal control group	-	127.270 ± 3.382	187.160 ± 5.192	1788.910 ± 47.413	462.210 ± 29.221
U27 Tumot Model group	-	112.369 ± 4.029^*^	166.252 ± 8.568^*^	1713.961 ± 49.607	440.741 ± 18.023^*^
CTX group	0.3	74.296 ± 13.993^*△^	99.052 ± 23.571^*△^	1373.506 ± 134.165^*△^	401.114 ± 15.965^*△^
ASMq.L group	2	120.720 ± 6.131^△▲^	174.750 ± 10.242^*△▲^	1760.940 ± 141.710^*△▲^	434.980 ± 18.858^*▲^
ASMq.M group	4	128.900 ± 4.822^△▲^	199.030 ± 9.627^*△▲^	1905.760 ± 79.073^*△▲^	440.190 ± 22.025^*▲^
ASMq.H group	8	136.640 ± 7.524^*△▲^	187.810 ± 11.047^△▲^	1991.600 ± 167.884^*△▲^	448.740 ± 22.692^▲^

Notes:^*^ *P* < 0.05 compared with Normal control group;^△^ *P* < 0.05 compared with U27 tumor model group;^▲^ *P* < 0.05 compared with CTX group

### Effects of ASMq on the tumor inhibition rates and vital organ indexes of the U27 tumor mice

The thymus, spleen, and liver indexes of the U27 tumor model group are lower than those of the normal control group (*p* < 0.05). In addition, the thymus, spleen, and liver indexes of the CTX group are decreased significantly (*p* < 0.05). The thymus, spleen, and kidney indexes of the low-dose ASMq group are also decreased significantly (*p* < 0.05). Likewise, the thymus, spleen, and kidney indexes of the medium-dose ASMq group are decreased (*p* < 0.05), while the thymus, spleen, and kidney indexes of the high-dose ASMq group are decreased significantly (*p* < 0.05). Compared to the U27 tumor model group, the thymus, spleen, and liver indexes of the CTX group are decreased significantly (*p* < 0.05), while the kidney indexes of the CTX group are increased (*p* < 0.05). Furthermore, the thymus, spleen, and kidney indexes of the low- and medium-dose ASMq groups as well as the spleen and kidney indexes of the high-dose ASMq group are decreased (*p* < 0.05). Compared to the CTX group, the thymus, spleen, and liver indexes of all ASMq groups are increased significantly (*p* < 0.05), while the kidney indexes of the ASMq groups are decreased (*p* < 0.05), as shown in Table 3.

**Table 3:** ASMq’s effect on the important organ index of in each group mice (*n* = 8, $\overline{x}\pm s$)

Group	Dosage/(g/kg)	Thymus index	Spleen index	Liver index	Kidney index
Normal control group	-	4.032 ± 0.104	5.929 ± 0.153	56.676 ± 1.570	14.637 ± 0.813
U27 Tumot Model group	-	3.578 ± 0.134^*^	5.288 ± 0.113^*^	54.585 ± 2.045^*^	14.034 ± 0.619
CTX group	0.3	2.702 ± 0.360^*△^	3.590 ± 0.599^*△^	50.088 ± 2.003^*△^	14.685 ± 0.812^△^
ASMq.L group	2	3.641 ± 0.230^*△▲^	5.262 ± 0.189^*△▲^	52.956 ± 1.934^▲^	13.122 ± 0.815^*△▲^
ASMq.M group	4	3.749 ± 0.203^*△▲^	5.780 ± 0.167^*△▲^	55.393 ± 2.520^▲^	12.802 ± 0.797^*△▲^
ASMq.H group	8	3.712 ± 0.156^*▲^	5.100 ± 0.135^*△▲^	54.026 ± 2.271^*▲^	12.196 ± 0.503^*△▲^

Notes:^*^ *P* < 0.05 compared with Normal control group;^△^ *P* < 0.05 compared with U27 tumor model group;^▲^ *P* < 0.05 compared with CTX group

The tumor inhibition rates of the CTX group and the ASMq.L group, ASMq.M group, and ASMq.H are approximately 72.21, 31.27, 60.53, and 51.94%, respectively. Compared to the U27 tumor model group, the tumor masses of the CTX group and all of the ASMq groups are decreased significantly (*p* < 0.05). In contrast, compared to the CTX group, the tumor masses of all of the ASMq groups increased (*p* < 0.05), as shown in Table 4.

**Table 4:** ASMq’s effect on the tumor growth of each group mice (*n* = 8, $\overline{x}\pm s$)

Group	Dosage/(g/kg)	Tumor weight/mg	Tumor inhibition rate/%
Normal control group	-	0.000 ± 0.000	-
U27 Tumot Model group	-	2102.891 ± 209.690	-
CTX group	0.3	584.273 ± 507.625^△^	72.21
ASMq.L group	2	1445.350 ± 327.236^△▲^	31.27
ASMq.M group	4	829.930 ± 283.190^△▲^	60.53
ASMq.H group	8	1010.540 ± 179.564^△▲^	51.94

Notes:^△^ *P* < 0.05 compared with U27 tumor model group;^▲^ *P* < 0.05 compared with CTX group

### Effects of ASMq on the histological sections of the U27 tumor mice

**Fig. 1:**
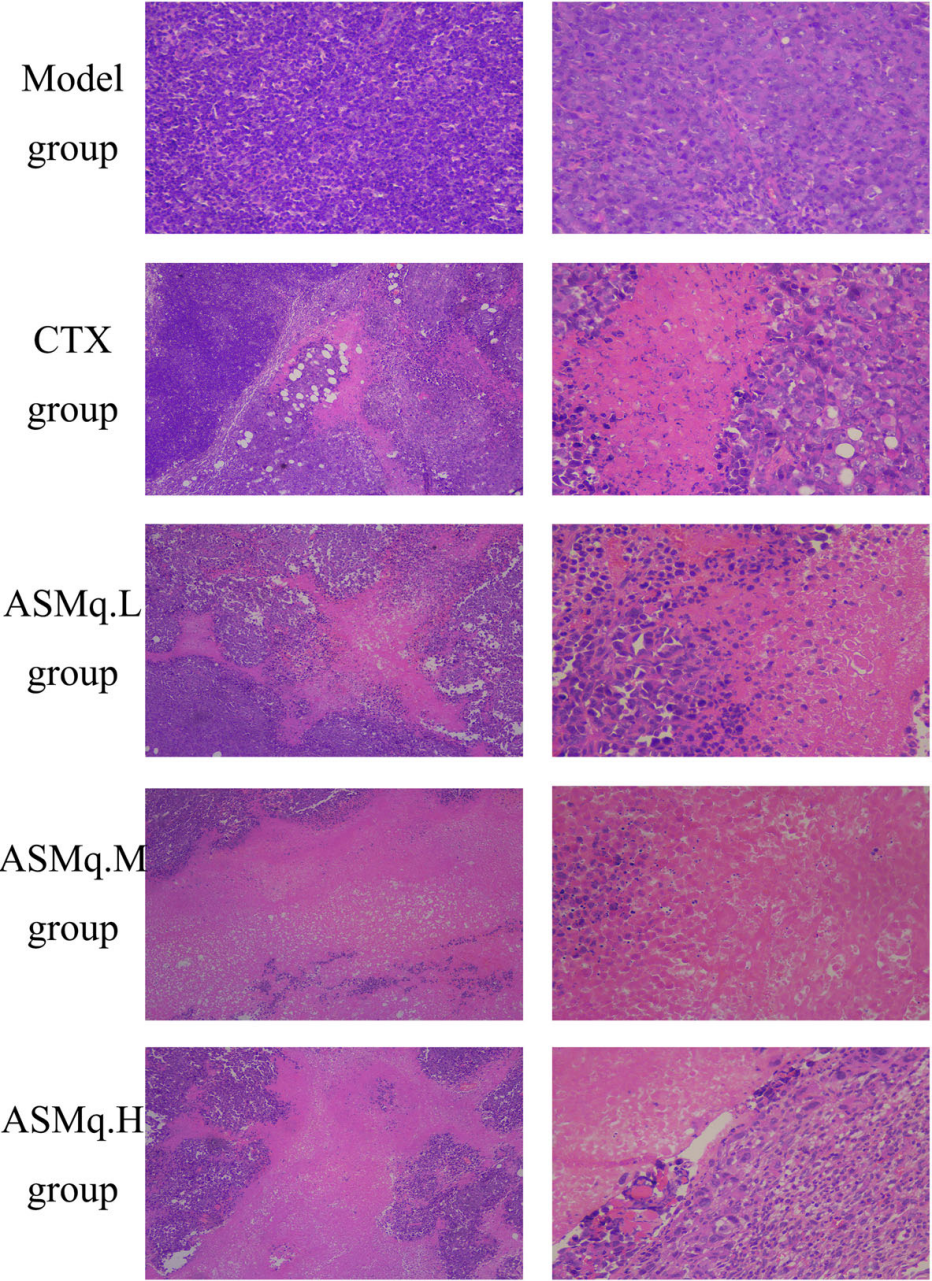
Pathological examination of mice tumor tissues

The tumors in the U27 model group exhibited invasive growth and rapid diffusion with high degrees of tumor cell proliferation. The cells were uneven in size and disordered, with different shades, large nuclei, less cytoplasm, visible nuclear mitosis, light edema, and infiltrated interstitial fluid containing a few inflammatory cells, as shown in Fig. 1 (U27 Tumor Model group). After the CTX treatment, the tumor cells exhibited unclear structures with regional necrosis, clear cell edema, and a large number of disintegrated granular cells, as shown in Fig. 1 (CTX group). After the low-dose ASMq treatment, a large number of malignant cells surrounded the necrotic tissue, with edema, nuclear condensation, different shades, and clear interstitial edema, as shown in Fig. 1 (ASMq.L group). After the medium-dose ASMq treatment, the tumor cells displayed large areas of necrosis with slight liquefaction. In addition, the cells at the borders of the necrotic tissue disintegrated into granular cells with interstitial edema, as shown in Fig. 1 (ASMq.M group). After the high-dose ASMq treatment, the tumor cells demonstrated regional necrosis, with shrunken cell volumes, nuclear condensation, and clear interstitial edema occasionally infiltrated with inflammatory cells, as shown in Fig. 1 (ASMq.H group).

### Effects of ASMq on TGF-β1 and TNF-α protein expression in the tumor tissues of the U27 model mice

**Fig. 2:**
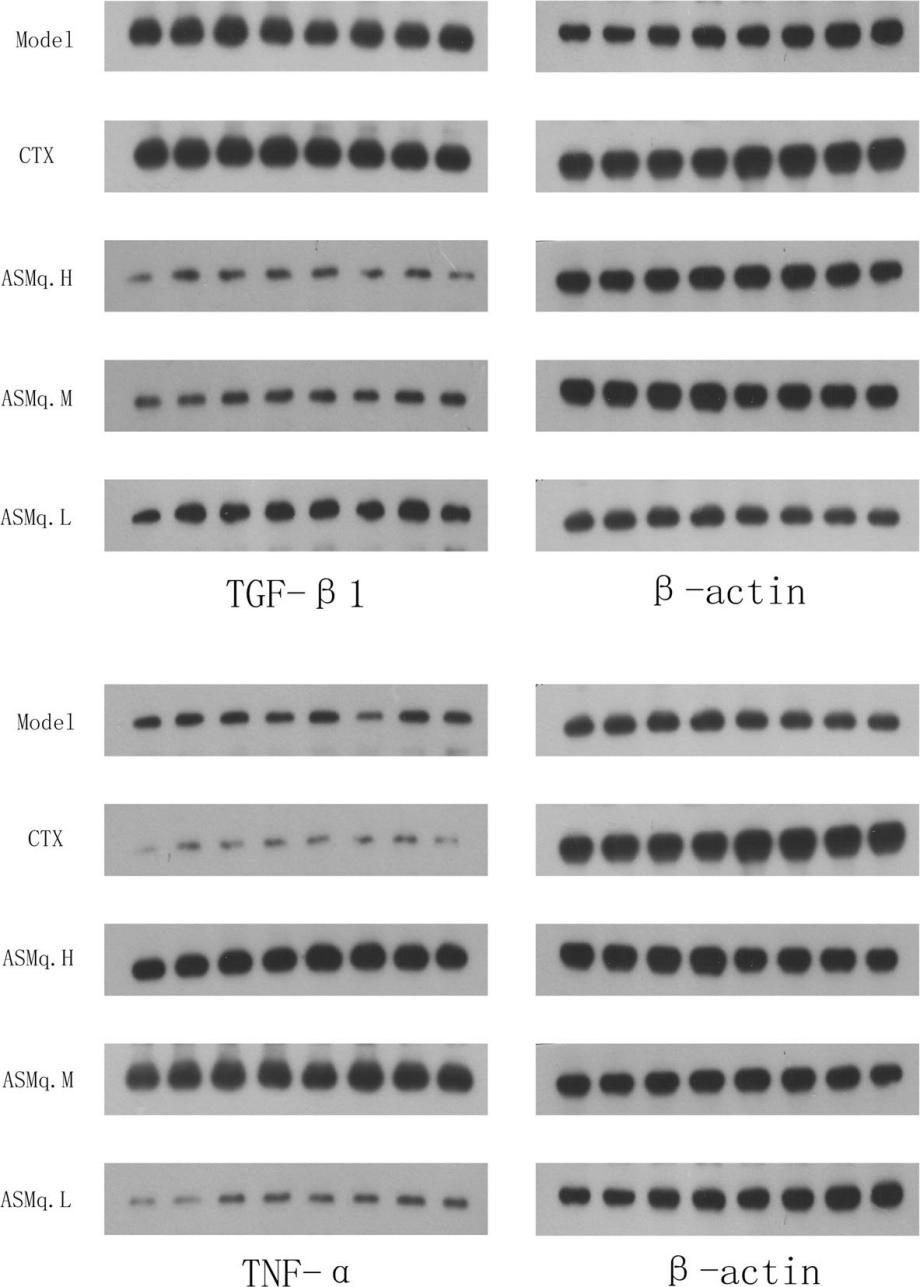
Expression of TGF-β1 and TNF-α in tumor tissues of mice in each group

The TGF-β1 and TNF-α expression levels of the tumor tissues were determined via the Western blot method. The quantitative results are shown in Table 5. Compared to the U27 tumor model group, the CTX group demonstrates significantly reduced TGF-β1 and TNF-α protein expression levels (*P* <0.05). In addition, the low-, medium-, and high-dose ASMq groups all demonstrates decreased TGF-β1 protein expression levels and significantly increased TGF-α protein expression levels (*p* < 0.05). Compared to the CTX group, the TGF-β1 expression levels of the ASMq groups are decreased (*p* < 0.05), while the TGF-α expression levels of the ASMq groups are increased (*p* < 0.05). as shown in Fig. 2

**Table 5:** ASMq’s expression on content of TGF-β1 and TNF-α in the tumor tissues of each group mice (*n* = 8, $\overline{x}\pm s$)

Group	Dosage/(g/kg)	TGF-β1	TNF-α
U27 Tumot Model group	-	1.4325 ± 0.1715	0.6783 ± 0.0964
CTX group	0.3	1.0561 ± 0.1185^△^	0.3370 ± 0.0377^△^
ASMq.L group	2	0.8537 ± 0.1090^△▲^	0.6948 ± 0.0711^▲^
ASMq.M group	4	0.5770 ± 0.0889^△▲^	1.2140 ± 0.1722^△▲^
ASMq.H group	8	0.5976 ± 0.0864^△▲^	1.0199 ± 0.1093^△▲^

Notes:^△^ *P* < 0.05 compared with U27 tumor model group;^▲^ *P* < 0.05 compared with CTX group

## Discussion

In recent years, numerous studies concerning the antitumor effects of ASMq have been conducted. In vitro studies are indicated that ASMq could inhibit the cell proliferation of breast cancer, liver cancer, lymphoma, and cervical cancer cells as well as promotes apoptosis [18–21]. However, very few reports concerning the in vivo mechanisms of ASMq exist. Our research team has been investigating the in vivo anti-tumor mechanisms of ASMq for a long time. By studying the in vivo mechanisms of ASMq, we found that ASMq shows a certain levels of therapeutic effects on EAC and S180 tumor cell [22–24]. In order to investigate the in vivo antitumor effects of ASMq on the U27 tumor mouse model, this study focused on the tumor inhibition rate, tumor histopathology, and TGF-β1 and TNF-a protein expression levels of tumor tissues obtained from the U27 tumor model group, CTX group, and low-, medium-, and high-dose of ASMq groups.

According to the results, after the administration of ASMq via gavage, the thymic and splenic weights of all the experimental groups are increased. In addition, the hepatic weights of the medium- and high-dose groups are also increased. The increases in the spleen and thymus indexes reflected enhanced T lymphocyte proliferation and improved immune function. Usually, the anti-tumor effects of Chinese herbal medicine are associated with immune function regulation [25, 26]. According to Chinese cancer research standards, herbal medicine is only considered to be effective as an anti-cancer treatment when the tumor inhibition rate is higher than 30% [27, 28]. The maximum tumor inhibition rate of ASMq within the tested dosage range was not as high as that of the CTX group; however, both of these substances yielded tumor inhibition rates greater than 30%, indicating that CTX and ASMq could both inhibits varying degrees of U27 tumor growth. Furthermore, the HE staining of the tumor tissue revealed necrosis and various cell sizes in the U27 model group and improved lesions in the CTX and ASMq groups. However, the CTX and ASMq groups still exhibited some areas of necrosis and edema. These results further confirmed the inhibitory effects of ASMq on U27 tumors.

TGF-β is a multifunctional cytokine that exists in a wide variety of normal and transformed cells. In fact, almost all cells are capable of producing TGF-β and expressing its receptors [29, 30]. TGF-β1 is a key factor in the regulation of the tumor epithelial-mesenchymal transition (EMT). TGF-β1, which is involved in cell proliferation and differentiation as well as the formation of the extracellular matrix, can promote tumor growth [31, 32]. According to previous studies, TGF-β1 could reduce the activity of NK cells and promote tumor cell proliferation and differentiation. Based on previous researches, high expression levels of TGF-β1 has been observed in a variety of tumors [33, 34]. According to the results of this study, the tumor tissues obtained from the U27 model group demonstrated significantly higher levels of TGF-β1 expression than the CTX group and the low-, medium-, and high-dose ASMq groups. These findings are consistent with the high expression rates of TGF-β1 in various malignant tumors. The decreased expression of TGF-β1 in the CTX and ASMq groups possibly relating to its downregulation by CTX and ASMq effects. Such as, after treating liver cancer in mice with tnshinone (Tan) IIA nanoparticles, significant tumor necrosis occurred and TGF-β1 expression was decreased, indicating that the anti-tumor effects of Tan IIA could function by inhibiting TGF-β1 expression [35]. Interestingly, some relating researches shows that TGF-β could directly or indirectly inhibits the production of TNF-α [36, 37], indicating that low concentrations of TGF-β were associated with high concentrations of TNF-α. TNF-α induces the tumor immune response and cell differentiation, promotes the production of a variety of cytokines by mononuclear cells and T cells, and induce tumor cell apoptosis and directly kill tumor cells [38–40]. In our study, the TNF-α protein expression levels of the low-, medium-, and high-dose ASMq groups are significantly higher than those of the U27 model group and CTX group. Recent studies shows that the anti-tumor effects of TNF-α were primarily enhanced by the body’s cellular and humoral immune functions, inhibiting tumor angiogenesis and directly inducing tumor cell apoptosis [41, 42]. Serum TNF-α and IL-12 levels has been reported to be increased in tumor-bearing mice treated with astragalus polysaccharides (APS)-induced mature dendritic cell (DC) vaccine therapy, suggesting that the anti-tumor effects of this therapy could stimulates the immune cells of tumor-bearing mice to produce anti-tumor cytokines TNF-α and IL-12 [43]. Our research results also suggests that ASMq shows anti-tumor effects through stimulating immune cells to produce anti-tumor cytokines.

## Conclusions

In summary, ASMq demonstrates anti-tumor effects and downregulates the expression of TGF-β1 and upregulates the expression of TNF-α in vivo. The results of this study provides a scientific basis for the clinical application of ASMq in the treatment of cervical cancer.

## Abbreviations

ASMq: Abnormal Savda Munziq
ASMq.H: Abnormal Savda Munziq high dose
ASMq.L: Abnormal Savda Munziq low dose
ASMq.M: Abnormal Savda Munziq medium dose
CTX: Cytoxan
